# A new family with spastic paraplegia type 51 and novel mutations in AP4E1

**DOI:** 10.1186/s12920-021-00980-5

**Published:** 2021-05-18

**Authors:** Izabela Winkler, Paweł Miotła, Monika Lejman, Aleksandra Pietrzyk, Magdalena Kacprzak, Marcin Kubiak, Agnieszka Sobczyńska-Tomaszewska, Maciej Skrzypczak, Ilona Jaszczuk

**Affiliations:** 1 2nd Department of Gynaecology, St. Johns Centre Oncology, Lublin Oncology Centre, 7 Jaczewski Street, 20-090 Lublin, Poland; 2 2nd Department of Gynaecology, Lublin Medical University, 8 Jaczewski Street, 20-954 Lublin, Poland; 3 Department of Paediatric Haematology, Oncology and Transplantology, Children Clinical Hospital, II Dept. of Paediatrics, Medical University, A. Gebali 6, 20-093 Lublin, Poland; 4 MedGen Medical Centre, Wiktorii Wiedeńskiej 9a Street, 02-954 Warsaw, Poland; 5 Department of Surgery, St. Johns Centre Oncology, 7 Jaczewski Street, 20-090 Lublin, Poland; 6 Department of Cancer Genetics with Cytogenetic Laboratory, Medical University of Lublin, Radziwiłłowska 11, 20-080 Lublin, Poland

**Keywords:** Genetic disorders, AP4E1, Spastic paraplegia, Neurological disorders, Next-generation sequencing, Psychomotor retardation, Cerebral palsy

## Abstract

**Background:**

Autosomal recessive mutations in the AP-4 (adaptor protein complex 4) complex subunit ϵ − 1 (AP-4E1) gene on chromosome 15q21.2 are known to cause spastic paraplegia 51 (SPG51). The exact phenotype of SPG51 remains poorly characterized, because only a few families have been reported as carriers of the mutation. In addition, a previous study identified an autosomal dominant mutation in the *AP4E1* gene as being associated with persistent stuttering. The aim of the current study was to characterize the phenotype of a paediatric patient with an identified novel *AP4E1* mutation presenting with significant psychomotor retardation, intellectual disability and paraplegia.

**Methods:**

Magnetic resonance imaging was used to identify hypoplasia of the corpus callosum. The DNA sample was tested using multiplex ligation-dependent probe amplification (MLPA) and array comparative genomic hybridization (aCGH). In addition, next-generation sequencing (NGS) was performed using the patient’s DNA, and Sanger sequencing was performed using that of his family members.

**Results:**

The phenotype was identified to be associated with a novel pathogenic variant c.942_943 + 3delinsCC in the *AP4E1* gene. The patient manifested severely delayed psychomotor development, impaired global physical development and general illness. Movement disorders were evident during the neonatal period.

**Conclusions:**

The present study identifies a previously unknown disease-inducing *AP4E1* gene mutation.

## Background

Spastic paraplegia-51 (SPG51; OMIM # 613744) is an autosomal recessive neurodevelopmental disorder that is characterized by the degeneration and dysfunction of the corticospinal and spinocerebellar tracts. Hypotonia in the neonatal period can progress to spasticity, hypertonia and severe intellectual disability, which is characterized by underdeveloped or non-existent speech. Moreover, cognitive and behavioural dysfunctions are notable [1, 2]. Spastic paraplegia is often confused with cerebral palsy. However, spastic diplegia or quadriplegia are not associated with birth order or parental age, while the age of the parents is associated with congenital hemiplegia and dystonic/athetoid cerebral palsy [3].

AP-4 complex subunit ϵ − 1 (AP-4E1) is involved in recognizing and binding sorting signals that are tyrosine-based and located within cytoplasmic cargos. In addition, AP-4E1 is able to recognize various sorting signals. Previous studies suggested that AP-4E1 facilitates the sorting of proteins to the basolateral membrane in epithelial cells and is involved in the establishment of effective somato-dendritic protein asymmetric localization in neurons [1–4].

AP-4-associated hereditary spastic paraplegia (HSP) is a group of neurodegenerative disorders that manifest as complex and progressive spastic paraplegia. Defects associated with HSP are typically identified during infancy or early childhood. Early-onset hypotonia becomes a progressive lower-extremity spasticity over time, and the patients affected by this disease are unable to walk or stand. Further progression of the spasticity affects the upper extremities, which leads to spastic tetraplegia. Additional complications include bladder and bowel dysfunction, dysphagia, foot deformities and contractures. In total, ~ 50% of patients experience seizures, and spastic paraplegia is associated with congenital microcephaly and global developmental delay. Furthermore, speech development is delayed, and speech is often absent [1, 2, 5].

The aim of this study was to characterize a novel mutation in the *AP4E1* gene that is correlated with the phenotypic variability of affected patients.

## Methods

The study was approved by local Ethics Committee. We obtained written informed consent for publication of the case file and clinical details from the patient’s parents. The comprehensive evaluation in the study relied on a detailed clinical history and the review and analysis of past medical records. The paediatric patient was the fourth child of non-consanguineous parents of Polish nationality. The boy was born at term (gestational week 41) after an uneventful pregnancy. His weight at birth was 4,100 g (> 99.9 percentile), his length was 55 cm (99 percentile), and his head circumference was 36 cm (85–90 percentile). The proband was delivered by caesarean section due to incipient foetal distress. His brother showed a similar history and had spastic paraplegia, while the other two siblings were healthy. Serious psychomotor retardation and general illness were evident in the neonatal period. In addition, dysmorphic features were noted, including periodic bilateral ptosis, a wide nasal ridge and long nose, an inverted full upper vermillion and short philtrum, marked ear antihelix and hypomimia of the face. No internal organ abnormalities were found following physical examination and ultrasound assessment. Lack of head control, eye contact and social smiling, in addition to marked axial hypotonia with bouts of hypertonia in the extremities, were identified on neurological examinations. No additional abnormalities were identified in the first six months by the physicians or the parents. Hyperplastic corpus callosum was diagnosed by magnetic resonance imaging, but hyperammonaemias, mitochondrial fatty acid beta-oxidation disorders, aminoacidopathies, glycosylation congenital disorders, neurotransmitter disorders and organic acidurias were excluded. Occasional ptosis excluded mitochondrial disease. Spastic diplegia was identified on neurological assessment at 2 years and 6 months of age. Therefore, treatment with botulinum toxin was undertaken. The male karyotype, array comparative genomic hybridization (aCGH) and multiplex ligation-dependent probe amplification (MLPA) were normal. At the last examination, the paediatric patient (age, 12 years) was 128 cm tall (< 3rd percentile), weighed 37 kg (25th percentile) and had a head circumference of 51 cm (< 3rd percentile). He could not stabilize his neck or torso, nor could he vocalize or rotate. His arm and leg movements were uncoordinated and restless. The proband could not smile and showed no response to loud noise. The parents, however, did not report seizures. The proband could balance on his hands and knees, and movements were possible with the aid of a rear balustrade. Physiotherapy improved his lower limb muscle tone. Examination did not indicate ptosis, and Babinski's bilateral sign was not evident.

Blood was collected, and DNA was extracted for further examinations. MLPA was performed as the initial diagnostic step. To perform MLPA, a P106-B2 (MRC Holland) kit was used. Next, aCGH (CytoSure Constitutional v3, 8 × 60 k; Oxford Gene Technology; GRCh37/hg19) was performed. The results of both test were normal. Further diagnostic procedures was performed using the NGS targeted SeqCap EZ (Nimle Gen) kit. For NGS analysis, the panel of 219 genes correlated with intellectual disability that was designed by the MedGen laboratory was used. The enriched DNA libraries were sequenced by the Illumina MiSeq instrument. The average depth of coverage for the AP4E1 gene was 100% per 30x.

Additionally, Sanger sequencing was performed for the patient and his family (parents and siblings).

The following in silico prediction software programs were used to assist with interpretation of the pathogenicity of detected variant: Alamut visual v2.9.0 (Interactive Biosoftware, SOPHiAGENETICS, CH-1025 Saint Sulpice, Switzerland); 1000 Genomes (http://www.l000genomes.org), which was used to verify the presence of the variant in control populations; the Exome Variant Server (http://evs.gs.washington.edu/EVS); the Exome Aggregation Consortium (http://exac.broadinstitute.org); and gnomAD (http://gnomad.broadinstitute.org/).

We deposit the sequences in a publicly available data repository (ClinVar), the accession number is VCV001064645.1.

## Results

**Fig. 1:**
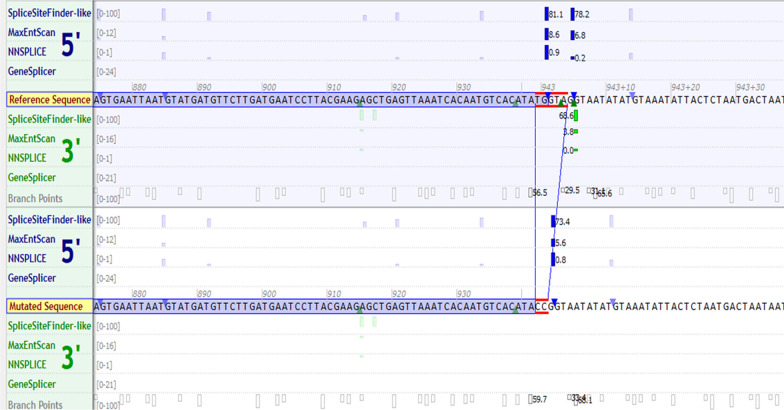
In silico prediction of the c.942_943 + 3delinsCC (3delTGGTAinsCC) homozygous mutation of the AP4E1 gene in the proband with spastic paraplegia type 51. According to NC_000015.9, NM_001252127.2, NM_007347.5 the sequence is CACATATGGTA

**Fig. 2:**
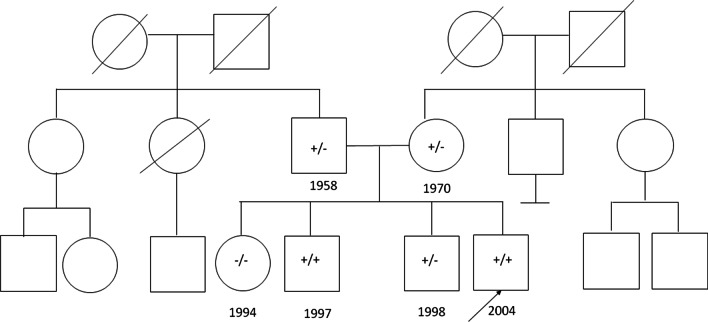
Family pedigree. The mutation status of each individual is presented. Three deletion status are indicated: (i) −/−, wild-type; (ii) +/−, unaffected carrier; and (iii) +/+, affected carrier. Year of birth is shown below square or circle

NGS examination revealed the mutation c.942_943 + 3delinsCC in the *AP4E1* gene (Fig. 1) located in the distal part of exon 8 and spanning two exonic and three intronic nucleotides, including a splicing donor site. Bioinformatic analysis using Alamut software predicted a new donor site, which resulted in a frameshift mutation introducing the premature termination of the codon after three nucleotides. It is also possible that the mutation may affect the splicing process (not examined in this study). The proband was homozygous for this mutation according to NGS. Sanger sequencing was used to confirm the presence of the variant in the paediatric patient and the following family members: his brother, who had spastic paraplegia; his other healthy siblings; and his parents (Fig. 2). The mutation c.942_943 + 3delinsCC in the *AP4E1* gene identified in the present study was not reported in the ClinVar (ncbi.nlm.nih.gov), LOVD (lovd.nl), ExAC (gnomad.broadinstitute.org) and dbSNP (ncbi.nlm.nih.gov/snp) databases. Therefore, this variant is considered a novel pathological variant that has not been previously reported. Moreover, the proband was heterozygous for the mutation p.Pro311Arg in the *SMARCA4* gene. This mutation was registered in the dbSNP database (rs759754994) but was not reported in the ClinVar and LOVD databases. The *SMARCA4* mutation was confirmed in the patient using Sanger sequencing, and his father was heterozygous. The presence of the *SMARCA4* mutation in the unaffected father suggests a non-pathogenic nature of the mutation. In the other siblings, mutations in the *SMARCA4* gene were not found; in particular, pathogenic variants in the *SMARCA4* gene are correlated with Coffin-Siris syndrome (CSS4, OMIM#614609).

## Discussion

The present study reported the case of a teenage boy with significant developmental delay, primary microcephaly and pyramidal-extrapyramidal tetraparesis. Cerebral palsy was initially diagnosed prior to genetic counselling. NGS was performed, and a disease-inducing mutation was found in the *AP4E1* gene encoding the AP-4 adaptor protein complex subunit ϵ − 1. To the best of our knowledge, the current study is the first report confirming the pathogenicity of the c.942_943 + 3delinsCC mutation.

According to Abou Jamra et al. [1], AP4 complex-mediated trafficking is crucial for brain development. In addition, this protein is involved in the formation of clathrin- and non-clathrin-coated vesicles and in integral membrane protein sorting. AP4 consists of 2 large chains, β-4 (AP4β1) and ϵ − 4 (AP4ϵ1); the medium chain, µ-4 (AP4µ1); and the small chain, σ-4 (AP4σ1) [5, 6]. Knock-out mice homozygous mutants exhibit abnormal white blood counts, enlarged lateral ventricles, a smaller corpus callosum and hypoferremia [6]. Additional mutations in the AP4E1 gene have been discovered. To date, ~ 80 individual mutations have been reported and included in the International Registry and Natural History Study of AP-4-Related Hereditary Spastic Paraplegia (updated 5–20-18). Considering the type of mutation (3delTGGTAinsCC), which induces a frameshift mutation and occurs in the proximity of a splicing site, it can be predicted that the identified mutation may affect the splicing process. Bioinformatic analysis showed that the detected variant may cause a change in the acceptor site, which further indicates its pathogenic nature. The homozygous indel mutation was found in another member of the family, the affected brother of the proband.

The parents were heterozygous for this mutation, one unaffected sibling was wild type, and the other unaffected sibling was heterozygous. Numerous case reports have been published describing patients with SPG51, and the clinical phenotype observed in the present study is consistent with the symptoms observed in an individual reported in a case study by Moreno De Luca et al. [2]. In the present study, the investigated subject never developed speech or independent ambulation. A comprehensive mutational analysis (including resequencing), as well as an analysis of *AP4E1* in other individuals with spastic paraplegia and in their families, should be undertaken to evaluate the frequency of this disorder.

Coffin-Siris syndrome, an autosomal condition, is caused by mutations in any of the following genes: *ARID1A (*AT Rich Interactive Domain 1A), *ARID1B* (AT Rich Interactive Domain 1B), *SMARCA4 (*SWI/SNF related, matrix associated, actin dependent regulator of chromatin, subfamily a, member 4), *SMARCB1* and *SMARCE1* [7]. The clinical features of Coffin-Siris syndrome include thick, arched eyebrows; a low anterior hairline; a broad-tipped nose; a short philtrum; columella below the alae nasi; simple posteriorly rotated ears; and a thick drooping lower lip [8]. Moreover, hyperkeratotic plaques and rough skin are also common features of this disease. In addition, the fingertips and feet are frequently broader than normal (Table 1). Furthermore, Poyhonen et al. [8] showed a short, thick corpus callosum confirmed by brain MRI.

**Table 1:** Clinical features of proband and his family

	Probant	Brother	Father
Intelligence	Severe intellectual disability	Deep intellectual disability	Normal
Speech development	A few words (He understands simple commands)	No speech development (He doesn’t understand commands)	Normal
Heigh	Short stature (< 3 pc)	Short stature (< 3 pc)	Medium stature (10 pc)
Microcephaly	Present	Present	No
Dysmorphic features			
Narrow Forehead	Yes	Yes	No
Ptosis	Yes	Yes	No
Bulbous nasal Tip	Yes	Yes	Yes
Full lips	Yes	Yes	Yes
Others	Large ears with raised ear lobes and thickening of the labrum	Large ears with raised ear lobes and thickening of the labrum	No
Neurologic problems			
Spastic paraplegia	Yes	Yes	No
Movement	Crawling, standing only with suport He doesn’t walk	Crawling, standing and walking only with support	Total
Others	Contractures Abduction contracture of the fingers Knee valgus Hypoplasia of the corpus callosum Several episodes of increased drowsiness, increased ptosis, decrease in muscle tone. Metabolic and mitochondrial diseases were excluded	Increasing contractures despite rehabilitation Abduction contracture of the fingers Knee valgus	Hypertension Non-insulin dependent diabetes

In the present case report, an autosomal dominant inheritance with variable expression and a possible paternal effect was identified. Family pedigree analysis indicated that the detected SMARCA4 mutation is most likely a benign variant. Over 94% of SMARCA4 gene missense changes are benign or are described as variants of uncertain significance (according to the TrapScore database).

## Conclusions

The present study indicates that the c.942_943 + 3delinsCC mutation in *AP4E1* is a pathogenic variant. Thus, early molecular diagnosis may significantly enhance the diagnostic process, clinical management and genetic counselling of patients suffering from HSP and their families. However, further studies are needed to investigate the causal association between the clinical features and the mutation. Future studies may clarify the associations between the observed clinical features and genetic mutation. Following the identification of an AP-4-associated HSP-causing pathogenic variant in one member of the family, prenatal testing and pre-implantation genetic diagnosis should be considered.

## Data Availability

Not applicable.

## Abbreviations

aCGH: Array comparative genomic hybridization
AP4: Adaptor protein complex 4
ARID1A: AT Rich Interactive Domain 1A
HSP: Hereditary spastic paraplegia
MLPA: Multiplex ligation-dependent probe amplification
NGS: Next-generation sequencing
SPG51: Spastic paraplegia 51
SMARCA4: SWI-/SNF-related, matrix-associated, actin-dependent regulator of chromatin, subfamily a, member 4
